# Soft palate fistula after radiofrequency ablation for primary snoring: a case report and literature review^☆^

**DOI:** 10.1016/j.bjorl.2017.02.005

**Published:** 2017-03-07

**Authors:** Lauren K. Reckley, Camilo Fernandez-Salvador, Edward T. Chang, Macario Camacho

**Affiliations:** Tripler Army Medical Center, Division of Otolaryngology-Head and Neck Surgery, Honolulu, United States

## Introduction

Snoring is a common complaint for patients seeking medical care. Snoring can disrupt the sleep of the bed partners or those sharing a room with a snorer. Although smartphone snoring apps have been used to determine the loudness and number of snores per hour,^1^ the bed partner is ultimately the main determinant as to what is and is not disruptive snoring. Snoring is usually due to fluttering of the soft palate as it vibrates against the pharyngeal walls. Although snoring can occur exclusively during nasal breathing,^2^ it more commonly occurs during oral breathing. Several techniques have been developed to treat snoring such as injection snoreplasty, pillar implants, Cautery Assisted Palatal Stiffening Operations (CAPSO), and radiofrequency ablation (RFA). Radiofrequency ablation has been effectively used in multiple surgical specialties. The mechanism of action for RFA is due to a low temperature, high frequency current causing local inflammation and eventually fibrosis and stiffening of the tissue.^3^ RFA of the soft palate is minimally invasive, carries relatively few complications, and aims to stiffen the soft palate. Complications of RFA include: mucosal ulceration, sloughing, mucosal crusting, hemorrhage, nerve damage, and palatal fistula.^4^ Our objective is to review the international literature to identify studies reporting findings for palatal fistulas as well as to present our case study in detail.

We first performed a search in PubMed to attempt to identify any published studies for RFA with soft palate fistula complication using the following search strategy: [(radiofrequency ablation) AND (snoring) AND {(fistula) OR (hole) OR (perforation)} AND (soft palate)]. An additional search was performed using the following: [(radiofrequency ablation) AND (complications) AND (palate)]. We subsequently summarized the findings for the patient who underwent his first RFA of the soft palate for primary snoring and had a temporary fistula.

The PubMed search strategy for RFA and palatal fistula yielded no case report discussing the complication of palatal fistula. Articles were identified in which complications after radiofrequency were presented as part of a larger case series; therefore, we summarize the 5 patients described amongst other patients and provide detailed findings from our case report.

Kezirian et al.^4^ performed a review of the incidence of complications for patients undergoing RFA of the upper airway to include the nose, soft palate, and tongue. We reviewed the articles they presented in their review^4^ and also performed our own review for articles, we identified no additional articles. We downloaded each article and summarize the detail to the extent possible within each publication. Boudewyns et al.^5^ reported one patient who received a single, midline ablation at 700 Joules (J) and developed a palatal fistula (out of 122 treatment sessions, 0.8% rate), which healed spontaneously. Rombaux et al.^6^ reported performing RFA with 4–6 palatal channels (settings not specified) in a patient who developed a “minor” soft palate fistula, noted 6 weeks after surgery; it was one out of 17 patients (a 5.9% rate), which resolved spontaneously. Sher et al.^7^ reported delivering 460 kilohertz between 60° and 90 °C to two patients who developed palatal fistulas (out of 113 patients, 1.9% rate) and had a 3–4 mm soft palate fistula that closed within several days without additional complications. Emery et al.^8^ delivered one midline ablation at 700 J and two lateral ablations at 300 J, for a total of 1200 J at 85 °C and described one case out of 43 patients (2.3% rate) who formed a palatal fistula in a 33-year-old woman with juvenile-onset (Type 1) diabetes *mellitus* which was primarily closed under local anesthesia. The fistula did not recur after primary closure. Previous palatal fistulas were reported as part of case series articles describing all complications of RFA. To our knowledge, no case report has been described with in-depth patient data, photo documentation, and description of the natural course after RFA for primary snoring; therefore, we report the findings from a patient treated at our tertiary medical center.

The Investigational Review Board (IRB) at our institution was contacted and this project does not meet the Federal definition of research [DHHS 45 CFR 46.102(d)] or the Federal definition of clinical investigation [FDA 21 CFR 50.3(c) and 56.102(c)].

## Case report

**Figure 1:**
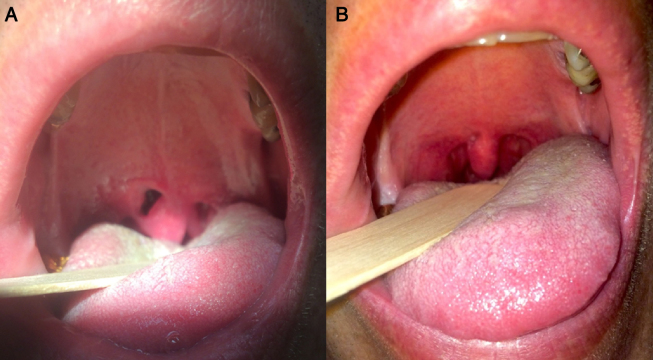
The palatal fistula is demonstrated in (A) and the healed fistula is demonstrated in (B).

Our patient is a 39-year-old male who presented to the otolaryngology-head and neck clinic with the complaint of heroic snoring and fatigue. His wife reported the snoring was 10 out of 10 (could be heard through a closed door and was significantly disruptive to her sleep). He had a history of hypothyroidism, but his thyroid labs were normal triiodothyronine 2.72 (reference range: 1.71–3.8) and free thyroxine 1.10 (reference range: 0.8–1.6) with 200 micrograms of levothyroxine. A sleep study was ordered, and he was diagnosed with primary snoring, with an apnea–hypopnea index of 3.0 events per hour, a lowest oxygen saturation of 91%, and periodic limb movements of 2.7 events per hour. On physical exam, the patient had a c-shaped nasal septal deviation^9^ (moderate in nature). Using the Inferior Turbinate Classification System,^10^ he had a grade 4 right inferior turbinate and a grade 3 left inferior turbinate. At the level of the choanae, there was 30% obstruction secondary to adenoid hypertrophy. Despite the nasal anatomical abnormalities, he denied any nasal obstruction. His tonsils were 2+ bilaterally, with redundant soft palate tissue, and was a Modified Mallampati grade 3. He had an abnormally long uvula, measuring approximately 1.8 cm. Because the snoring significantly bothered his wife, he elected for RFA of the soft palate. The procedure was performed in the operating room under general anesthesia. The Gyrus radiofrequency device was set to 81 °C and 700 J. The probe was passed in the midline and 1 cm into the paramedian locations bilaterally (2100 J total). The patient was seen one week following surgery and was doing well without fever, pain, or complication. He called the clinic and was seen two weeks postoperatively after he had developed a 5 mm × 10 mm palatal fistula which allowed the pharynx to be visualized (Fig. 1A). He noticed the palatal fistula because he could feel it with his tongue. He denied bleeding or pain without taking medication. The patient was not prescribed antibiotics as there were no signs of infection, and there was no literature supporting prophylactic antibiotic use for a similar case presentation. He was also advised that he may continue a regular diet but to be careful about having hard or crunchy food penetrate or abrade the fistula. The patient was observed for 4 additional weeks and then had complete closure of the fistula, after healing by secondary intention (Fig. 1B) and had a very small (2 mm) section of mucosal ulceration which was almost completely healed. Snoring based on bedpartner reports had significantly improved to 2 out of 10 on the visual analog scale by 8 weeks post-operatively (however, stated at later visits that the snoring is starting to recur).

## Discussion

Ideally, delivery of energy via radiofrequency ablation to muscular tissue induces the optimal efficacy from this procedure. Consequently, the parameters of energy delivery by the radiofrequency ablation devices typically require settings to detect the resistance found in muscle tissue. The energy based on muscle tissue resistance exceeds the allowable energy to overcome the resistance found in mucosal tissue. The area of maximal energy delivery encompasses one centimeter found at the tip of the wand of the radiofrequency hand piece.

As with any surgical procedure, the outcome is at least somewhat operator dependent. Therefore, the operator should incorporate every measure of precaution to ensure proper placement of the wand within the appropriate muscle tissue depth. Specific placement of the radiofrequency ablation wand can be challenging to assess, therefore we recommend using a dental mirror to look into the nasopharynx (via the intraoral route) during the RFA wand positioning, thereby ensuring the wand is not too deep. Placement of the tip of the RFA instrument too superficial or too deep can cause thermal injury to the mucosa. Based on proximity of the region of maximal delivery of radiofrequency energy to the mucosa, excess energy to mucosa appears a potential complication of this procedure. We hypothesize that radiofrequency damage to mucosa at a setting to ablate muscle tissue likely presents the potential for destructive ablation and development of a palatal fistula. If the three destructive ablation passes are performed within too close to one another, then there may be excessive tissue destruction, which in turn increases the likelihood of palatal fistula formation. Given that a palatal fistula is an open wound and the surrounding healthy tissue is well perfused, we did not prescribe antibiotics to this patient. However, it is possible that there may be a role for antibiotics in an immunocompromised patient (i.e. a human immunodeficiency syndrome patient or a diabetic patient).

In the international literature, we identified 5 out of 295 patients (1.7% rate) who developed palatal fistulas after radiofrequency ablation of the soft palate. Only one patient was treated with primary closure, and that patient had Type 1 diabetes *mellitus*. The primary closure was likely performed in the setting of the patient having Type 1 diabetes as it is known to cause impaired wound healing, especially if the glucose levels are poorly controlled. Aside from the one patient who underwent primary closure, the remaining four patients in the literature healed by secondary intention. In our patient, with a 5 mm × 10 mm palatal fistula, the palate healed by secondary intention within 6 weeks.

Overall, radiofrequency ablation of the soft palate is a minimally invasive procedure, generally limited to treating snoring and mild OSA. Radiofrequency ablation of the soft palate can also serve as an adjunct treatment, simultaneously performed with other procedures without adding significant morbidity. Additional research on radiofrequency ablation of the soft palate can help determine the frequency and severity of complications for this therapy as treatment for snoring and OSA.

## Conclusion

Based on published literature, palatal fistulas secondary to radiofrequency ablation occur in about 1.7% of the treatment sessions. We did not identify any patient with a persistent palatal fistula in the international literature; therefore, patients who are not immunocompromised should heal by secondary intention. Our patient was a primary snorer, treated with radiofrequency ablation, who had the largest palatal fistula reported in the literature (5 mm × 10 mm), and it resolved by secondary intention within 6 weeks postoperatively.

## Conflicts of interest

The authors declare no conflicts of interest.

## References

[1] Camacho M., Robertson M., Abdullatif J., Certal V., Kram Y.A., Ruoff C.M. (2015). Smartphone apps for snoring. J Laryngol Otol.

[2] Hsia J.C., Camacho M., Capasso R. (2014). Snoring exclusively during nasal breathing: a newly described respiratory pattern during sleep. Sleep Breath.

[3] Powell N.B., Riley R.W., Troell R.J., Li K., Blumen M.B., Guilleminault C. (1998). Radiofrequency volumetric tissue reduction of the palate in subjects with sleep-disordered breathing. Chest.

[4] Kezirian E.J., Powell N.B., Riley R.W., Hester J.E. (2005). Incidence of complications in radiofrequency treatment of the upper airway. Laryngoscope.

[5] Boudewyns A., Van De Heyning P. (2000). Temperature-controlled radiofrequency tissue volume reduction of the soft palate (somnoplasty) in the treatment of habitual snoring: results of a European multicenter trial. Acta Otolaryngol.

[6] Rombaux P., Hamoir M., Bertrand B., Aubert G., Liistro G., Rodenstein D. (2003). Postoperative pain and side effects after uvulopalatopharyngoplasty, laser-assisted uvulopalatoplasty, and radiofrequency tissue volume reduction in primary snoring. Laryngoscope.

[7] Sher A.E., Flexon P.B., Hillman D., Emery B., Swieca J., Smith T.L. (2001). Temperature-controlled radiofrequency tissue volume reduction in the human soft palate. Otolaryngol Head Neck Surg.

[8] Emery B.E., Flexon P.B. (2000). Radiofrequency volumetric tissue reduction of the soft palate: a new treatment for snoring. Laryngoscope.

[9] Teixeira J., Certal V., Chang E.T., Camacho M. (2016). Nasal septal deviations: a systematic review of classification systems. Plast Surg Int.

[10] Camacho M., Zaghi S., Certal V., Abdullatif J., Means C., Acevedo J. (2015). Inferior turbinate classification system, grades 1 to 4: development and validation study. Laryngoscope.

